# Supplementary figures and images for: Spatial and temporal trends in dung beetle research
Source: PeerJ. 2025 Feb 21;13:e18907. doi: 10.7717/peerj.18907 (PMC11849510; doi:10.7717/peerj.18907)

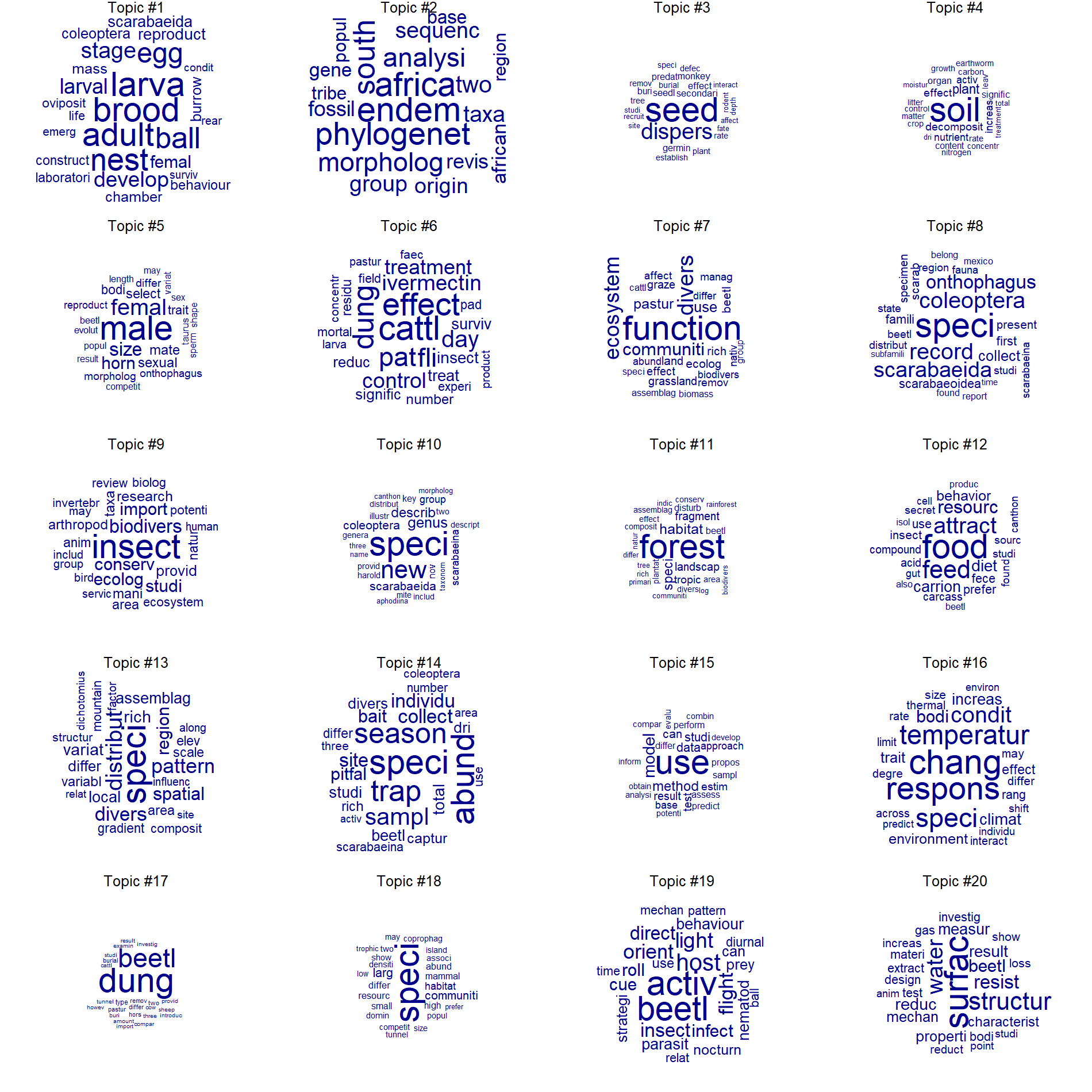

Supplement: Supplemental Information 1 — The corpus consists of articles relating to dung beetles published between 1933 and January. [file peerj-13-18907-s001.png]
